# Proteomic Characterization of the Cellular Effects of AhR Activation by Microbial Tryptophan Catabolites in Endotoxin-Activated Human Macrophages

**DOI:** 10.3390/ijerph181910336

**Published:** 2021-09-30

**Authors:** Katharina Walter, Henning Grosskopf, Isabel Karkossa, Martin von Bergen, Kristin Schubert

**Affiliations:** 1 Department of Molecular Systems Biology, Helmholtz Centre for Environmental Research, 04318 Leipzig, Germany; katharina.walter@ufz.de (K.W.); henning.grosskopf@ufz.de (H.G.); isabel.karkossa@ufz.de (I.K.); martin.vonbergen@ufz.de (M.v.B.); 2 Institute of Biochemistry, Leipzig University, 04318 Leipzig, Germany

**Keywords:** AhR, macrophages, proteome, BaP, indoles, indole-3-acetic acid, indole-3-aldehyde, LPS

## Abstract

Sensing microbial tryptophan catabolites by the aryl hydrocarbon receptor (AhR) plays a pivotal role in host-microbiome homeostasis by modulating the host immune response. Nevertheless, the involved cellular processes triggered by the metabolites are mainly unknown. Here, we analyzed proteomic changes in macrophages after treatment with the tryptophan metabolites indole-3-acetic acid (I3AA) or indole-3-aldehyde (IAld), as well as the prototypic exogenous AhR-ligand benzo(a)pyrene (BaP) in the absence and presence of lipopolysaccharide (LPS) to identify affected cellular processes and pathways. The AhR-ligands regulated metabolic and immunologic processes in dependency of LPS co-stimulation. All investigated ligands time-dependently enhanced fatty acid β-oxidation. Differences due to the combination with LPS were observed for all three ligands. Additionally, oxidative phosphorylation was significantly increased by IAld and I3AA in a time and LPS-dependent manner. Immunoregulatory processes were affected in distinct ways. While BaP and I3AA up-regulated IL-8 signaling, IL-6 signaling was decreased by IAld. BaP decreased the inflammasome pathway. Thus, AhR-ligand-dependent regulations were identified, which may modulate the response of macrophages to bacterial infections, but also the commensal microbiota through changes in immune cell signaling and metabolic pathways that may also alter functionality. These findings highlight the relevance of AhR for maintaining microbial homeostasis and, consequently, host health.

## 1. Introduction

The Aryl hydrocarbon receptor (AhR) is a ligand-activated transcription factor that binds to endogenous metabolites and xenobiotic chemicals that feature an aromatic ring system, serving as an environmental sensor. It has recently gained emerging interest due to its safeguarding barrier function in the intestines [1]. In its inactive form, AhR is part of a cytosolic complex with heat shock protein 90 (HSP90), AH receptor-interacting protein (AIP), and Hsp90 co-chaperone p23 (P23) [2,3,4,5]. Upon ligand binding, AhR is released from the cytosolic complex and translocates to the nucleus, where it dimerizes with the aryl hydrocarbon receptor nuclear translocator (ARNT). The AhR-ARNT complex is then recruited to xenobiotic response elements (XRE), initiating target gene expression [6], e.g., CYP1A1, CYP1B1, IDO1, and TDO2, as well as the aryl hydrocarbon receptor repressor (AHRR), providing a negative feedback loop [7]. Most AhR-ligands are halogenated dioxins, polychlorinated biphenyls, or polyaromatic hydrocarbons (PAHs) [8]. Benzo(a)pyrene (BaP) is a prototypic, widespread PAH mainly originating from incomplete combustion that may occur during tobacco smoking [9,10,11].

Besides environmental contaminants, host metabolites of the Kynurenine and Serotonine pathways as well as microbiota-derived tryptophan metabolites serve as AhR-ligands and exert anti-inflammatory effects [12]. Two of the microbiota-derived ligands are indole-3-acetic acid (I3AA) and indole-3-aldehyde (IAld) [13,14,15], which can be found in human feces in concentrations of ~45 µmol/kg and 4 µmol/kg, respectively [16]. The commensal bacteria *Lactobacillus reuteri* produce both of them through the indole pyruvate route catalyzed by aromatic amino acid aminotransferase (ArAT) [15]. In patients with metabolic syndrome, the intestinal microbiota has a decreased capacity to generate tryptophan-derived AhR-ligands [16]. In that line, administration of the AhR-ligand 6-formylindolo[3,2-b]carbazole (FICZ) or *Lactobacillus reuteri*, which has a high capacity to produce tryptophan-derived AhR-ligands, led to an improvement of the metabolic impairments in a mouse model with genetically-induced metabolic syndrome [17]. Furthermore, indole metabolites up-regulate the interleukin-10 receptor expression on intestinal epithelial cells, reducing inflammatory processes and fostering intestinal homeostasis in mice and humans [18]. In that line, the transcription of Interleukin-22 (IL-22) is AhR-dependently increased in type 3 innate lymphoid cells (ILC3), which balance the mucosal response to allow mixed microbial communities and homeostasis on one side but protect from candidiasis on the other [15]. In contrast, the intestinal microbiota’s inefficiency to produce tryptophan-derived AhR-ligands and the resulting impaired IL-22 production is involved in the pathogenesis of inflammatory bowel disease [19].

Besides its expression in ILCs, the AhR is expressed in most intestinal immune cells and is of importance for their development, preservation, and function [20,21]. Intestinal macrophages are the largest pool of tissue-associated macrophages and, in general, the mononuclear phagocytes [22,23]. Macrophages of AhR-knockout (AhR^−/−^) mice produce higher amounts of proinflammatory cytokines (TNF, IL-6, IL-12), and these AhR^−/−^ mice are more sensitive to lipopolysaccharide (LPS)-induced septic shock [24,25]. Besides, treatment with BaP decreases the production of proinflammatory cytokines and increases the secretion of anti-inflammatory IL-10 in an AhR-dependent manner after pattern recognition receptor (PRR) activation in murine bone marrow-derived macrophages [26]. The decrease of inflammatory processes in LPS-activated monocyte-derived macrophages by BaP and FICZ has recently been shown to be partially dependent on non-genomic AhR-signaling, leading to a decreased RAC-alpha serine/threonine-protein kinase (AKT)-activity based on the ubiquitination of Rac1 [27].

While the effects of AhR activation by environmental stressors are well investigated, knowledge of the consequences of AhR activation by microbial metabolites in immune cells like macrophages is scarce. Thus, we aimed to investigate the cellular effects of the two tryptophan catabolites I3AA and IAld in human primary monocyte-derived endotoxin-activated macrophages using a global proteomics approach. We compared the modes of action to that of the well-described environmental contaminant BaP.

## 2. Materials and Methods

### 2.1. Cell Culture 

Buffy coats were obtained from healthy donors, who provided written informed consent, from the Institute of Transfusion Medicine, Leipzig, Germany, as approved by the local ethics committee (Ref.#079-15-09032015). Peripheral blood mononuclear cells (PBMC) were enriched from buffy coats by Ficoll (GE Healthcare, Danderyd, Sweden) gradient centrifugation. Primary monocytes were enriched from PBMC by adherence to the surface of cell culture plates for 30 min in RPMI 1640 (Life Technologies, Carlsbad, CA, USA) supplemented with Penicillin/Streptomycin (Sigma-Aldrich, St. Louis, MO, USA). Monocytes were collected and differentiated to macrophages by adding 5% heat-inactivated FBS (Biowest, Nuaillé, France) and 100 ng/mL recombinant human M-CSF (PeproTech, Hamburg, Germany) for 5 d, as described previously [28].

The differentiated macrophages were washed carefully with warm PBS and covered with 1 mM EDTA (Sigma-Aldrich, Hamburg, Germany) in PBS for 10 min. The same volume of RPMI-1640 + 3% FCS + 1% P/S was added, and the cells were carefully lifted from the plate and collected by centrifugation for 8 min at 250× *g*. 1.5 × 10^6^ cells were seeded in 2 mL of RPMI-1640 + 3% FCS + 1% P/S on 6-well plates.

After resting overnight, the media was exchanged, and the macrophages were stimulated with 2 µM BaP (Sigma Aldrich, Hamburg, Germany), 50 µM IAld (Carl Roth, Karlsruhe, Germany), or 50 µM I3AA (Carl Roth, Karlsruhe, Germany), each dissolved in dimethyl sulfoxide (DMSO) (Sigma Aldrich, Hamburg, Germany), with or without the addition of 100 ng/mL LPS-EB (InvivoGen, San Diego, CA, USA). An equal volume of DMSO (2 µL in 2 mL medium) was used for unstimulated control samples. After incubation for 2, 4, 16, or 24 h, the supernatant was recovered for TNF-ELISA, and the cells were lysed with 100 µL lysis buffer (8 M Urea (Merck, Darmstadt, Germany) in 20 mM HEPES (Sigma Aldrich, St. Louis, MO, USA) (pH = 8)) after washing 3 times with PBS. The cell lysate was recovered, sonicated for 2 min, and cleared by centrifugation (16.000 *g*, RT, 10 min). The protein concentration was determined by colorimetric assay (Pierce, Thermo Fischer Scientific, Waltham, MA, USA) at 660 nm.

### 2.2. Enzyme-Linked Immunosorbent Assay (ELISA)

TNF contents in cell culture supernatants were determined using the BD OptEIA^TM^ Human TNF ELISA Set (BD Biosciences, Franklin Lakes, NJ, USA) according to the manufacturer’s protocol.

### 2.3. Quantification of mRNA Expression Levels

The cells were treated for 6 h with ligands with or without LPS. Consecutively, the total RNA was isolated using the RNeasy kit (QIAGEN, Hilden, Germany). Single-stranded cDNA was synthesized using the High Capacity cDNA Reverse Transcription Kit (Thermo Fisher Scientific, Waltham, MA, USA). The qPCR was performed with a qPCR ABI 7500 Fast Real-Time PCR System (Applied Biosystems, Waltham, MA, USA) under usage of TaqMan™ Fast Advanced Master Mix (Thermo Fisher Scientific, Waltham, MA, USA). The following primers were used: (1) Hs00164383_m1, CYB1B1, FAM-MGB (2) Hs00174128_m1, TNFalpha, FAM-MGB (3) Hs00420895_gH, RPLPO, and FAM-MGB (all Thermo Fisher, USA). Fold changes were calculated by the ΔΔct-method relative to the unstimulated control with RPLP0 as the reference [29].

### 2.4. Sample Preparation for LC-MS/MS-Based Proteomics

For proteome analysis, 20 µg protein per sample was digested and labeled with tandem mass tags (TMT), as previously described [30,31]. In short, the volume of protein-containing lysate was adjusted to 100 µL with 100 mM triethylammonium bicarbonate (TEAB) (Sigma Aldrich, Hamburg, Germany). Proteins were reduced with 9.5 mM tris-(2-carboxyethyl)-phosphin (TCEP) (Sigma Aldrich, Hamburg, Germany) for 1 h at 55 °C, followed by alkylation with 17.05 mM iodoacetamide (IAA) (Merck, Darmstadt, Germany) for 30 min at room temperature in the dark. The samples were acidified with formic acid (Merck, Darmstadt, Germany). Then, 120 µL ACN (Merck, Darmstadt, Germany) was added to reach an organic solvent content higher than 50% (*v*/*v*). Proteins were loaded on 30 µg SpeedBead™ Magnetic Carboxylate modified particles (Sigma Aldrich, St. Louis, MO, USA). After an incubation time of 8 min, the samples were washed twice with 200 µL 70% (*v*/*v*) EtOH (Merck, Darmstadt, Germany) and once with 200 µL ACN (Merck, Darmstadt, Germany). For protein cleavage, Sequencing Grade Modified Trypsin (Promega, Fitchburg, WI, USA) in 100 mM TEAB was used in an enzyme:protein ratio of 1:50 overnight at 37 °C. The obtained peptides were labeled with 109 µg TMT10plex™ Isobaric Label Reagent (Thermo Fisher Scientific, Waltham, MA, USA) in ACN for 1 h. The reaction was quenched with 5% hydroxylamine (Thermo Fisher Scientific, Waltham, MA, USA) for 15 min. ACN was added to reach ≥ 95% (*v*/*v*) organic solvent, facilitating peptide binding to the beads. The differentially labeled samples of one biological replicate were combined. Bead-bound peptides were cleaned by washing with ACN two times. The peptides were eluted in two steps, first with 87% (*v*/*v*) ACN in 10 mM ammonium formate (pH 10) (Agilent Technologies, Santa Clara, CA, USA) and then with 2% (*v*/*v*) DMSO, resulting in two fractions per sample. Fractions were evaporated to dryness and reconstituted in 0.1% (*v*/*v*) formic acid (Merck, Darmstadt, Germany) directly before LC-MS/MS analysis.

### 2.5. LC-MS/MS

LC-MS/MS analysis of samples was performed on an UltiMate 3000 RSLCnano system (Dionex, Sunnyvale, CA, USA), online coupled to a Q Exactive HF mass spectrometer (Thermo Fisher Scientific, Waltham, MA, USA) by a chip-based electrospray ionization source (TriVersa NanoMate, Advion, Ithaca, NY, USA) as described before [32], with the exception that not the top 10 but the top 15 precursors were isolated and fragmented for peptide identification and quantification. In short, peptides were loaded on a trapping column (Acclaim PepMap 100 C18, 3 μm, nanoViper, 75 μm × 5 cm, Thermo Fisher Scientific, Waltham, MA, USA) and subsequently separated on an analytical column (Acclaim PepMap 100 C18, 3 μm, nanoViper, 75 μm × 25 cm, Thermo Fisher Scientific, Waltham, MA, USA) using a 169.5 min gradient of increasing CAN concentration in 0.1% formic acid.

### 2.6. Data Analysis

The LC-MS/MS raw data were deposited to the ProteomeXchange Consortium via the PRIDE [33] partner repository (identifier PXD025022) and were examined with ProteomeDiscoverer 2.2.0.388, performing the database search against the Uniprot Homo Sapiens RefSet (03/2019, 73,947 entries). An iterative workflow was applied as described before [34]. The following parameters were used: Maximum missed cleavages = 2, minimal peptide length = 6 amino acids, minimum number of peptide sequences = 2, peptide tolerance = 10 ppm, and FTMS MS/MS match tolerance = 20 ppm. For all searches, carbamidomethylation of cysteine and TMT labeling at Lys were set as fixed modification and TMT labeling on the N-terminus as dynamic modification. For the first search, protein N-terminal acetylation and oxidation of methionine were set as variable modifications additionally. For the second search, deamidation of Asn and Gln, carbamylation, and Gln -> pyro-Glu were added as dynamic modifications. For the third search, no additional modifications were added, but it was searched for semi-tryptic peptides. Identified peptides, proteins, and sites were filtered by a target-decoy approach applying an FDR ≤ 0.01 and using a reversed decoy database. 

Further statistical analyses were performed with R 3.6.0. The unstimulated samples containing only the DMSO amount used for all samples were set as control samples for the treatments with LPS, BaP, IAld, and I3AA, while the LPS-treated samples were used as controls for the co-stimulated samples. Fold changes (FCs) of the protein abundances compared with the respective controls were determined, Log2-transformed, and median-normalized. Reliable proteins had to be identified in at least three replicates. Based on the FCs, the significances were determined using the student’s *t*-test, defining *p*-value ≤ 0.05 to consider changes as significant. The quantified proteins were assigned to their cellular localization based on the Uniprot Homo Sapiens RefSet (03/2019, 73947 entries) by FunRich 3.1.3 [35,36]. The significance of enrichment of cellular localization was calculated based on hypergeometric testing with subsequent adjustment for multiple testing, according to Benjamini and Hochberg. Further pathway analyses based on the Log2-transformed FCs and p-values were performed applying Ingenuity Pathway Analysis (IPA) with the following settings: species: human, confidence: experimentally observed, tissue: dendritic cells and macrophages, network interactions included endogenous chemicals, and a *p*-value cutoff of 0.05 was defined. The significant enrichment of a pathway was assumed with an adjusted *p*-value ≤ 0.05 (based on Fisher’s exact *t*-test with subsequent adjustment for multiple testing according to Benjamini and Hochberg).

## 3. Results

**Figure 1:**
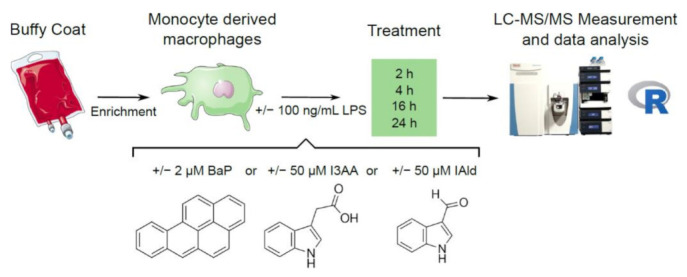
Experimental design. PBMCs were isolated from Buffy Coats and monocytes enriched. Afterward, these were differentiated to macrophages and stimulated with 100 ng/mL LPS or the AhR-ligands BaP (2 µM), I3AA (50 µM) and IAld (50 µM) with or without additional LPS for 2, 4, 16 and 24 h. Cell lysates were used to perform global proteomics and subsequent data analysis.

Previously, we and others have shown that AhR activation by exogenous ligands leads to anti-inflammatory effects in activated macrophages [26,27]. This study aimed to analyze global proteomic effects on cellular processes in macrophages induced by microbiota-derived AhR-ligands under inflammatory conditions in a time-dependent manner. Therefore, we stimulated macrophages with the microbial AhR-ligands indole-3-aldehyde (IAld) and indole-3-acetic acid (I3AA) and the exogenous ligand benzo(a)pyrene (BaP) with or without LPS and analyzed changes in protein abundances after 2, 4, 16, and 24 h (Figure 1). 

**Figure 2:**
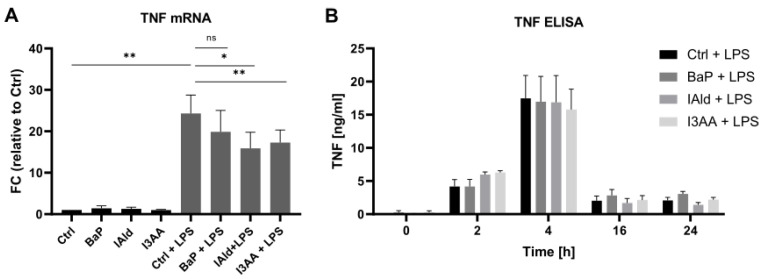
TNF expression and secretion. Macrophages were activated with 100 ng/mL LPS in the presence or absence of the AhR-ligands BaP, IAld, or I3AA. (**A**) The mRNA expression was determined by qPCR after 6 h of treatment. Data were normalized to RPLP0, and the gene expression level was calculated relative to the unstimulated control. Data are shown as mean ± SEM (*n* = 4). Significances were calculated by a two-sided, paired *t*-test (* *p* ≤ 0.05; ** *p* ≤ 0.01; ns, not significant). (**B**) Secreted TNF was determined by ELISA after 0, 2, 4, 16, and 24 h of treatment. Data are shown as mean ± SEM (*n* = 6 for 2 h and 4 h, *n* = 5 for 0 h, 16 h, and 24 h).

First, macrophage activation through stimulation with LPS in the presence of the AhR-ligands BaP, IAld, and I3AA was determined based on TNF expression and secretion. The expression level of TNF was determined by qPCR after 6 h. LPS-treatment induced an approximately 25-fold induction of TNF expression compared with the unstimulated sample. The co-treatment with IAld and I3AA reduced the TNF expression significantly (Figure 2A).

The secretion of TNF over 24 h of LPS-stimulation and exposure to AhR-ligands was determined by ELISA. As expected, exclusive treatment with LPS and the combination of LPS and the AhR-ligands led to an increase of the released TNF within the first 4 h (Figure 2B). No significant differences in the TNF release were observed due to exposure to AhR-ligands.

**Figure 3:**
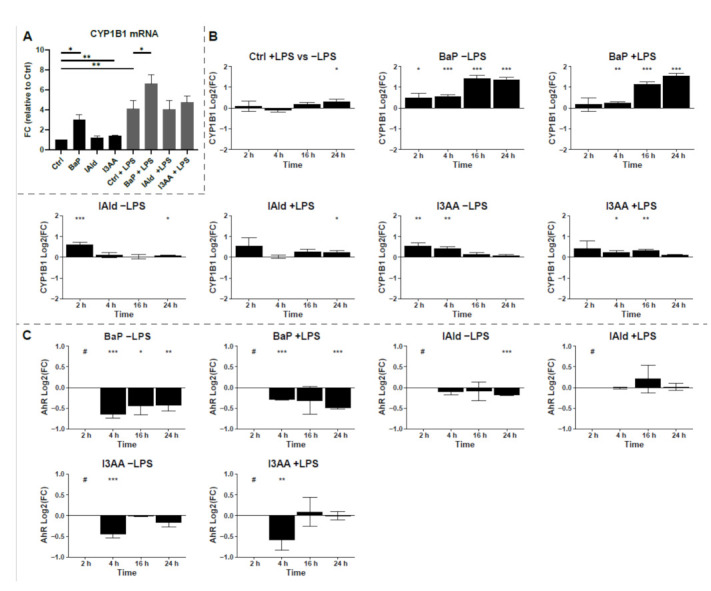
Induction of CYP1B1. (**A**) mRNA expression of CYP1B1 was determined by qPCR after 6 h of treatment. Data were normalized to RPLP0, and the gene expression level was calculated relative to the unstimulated control. Data are shown as mean ± SEM (*n* = 5). Significances were calculated by a two-sided, paired *t*-test. (**B**) Relative protein abundances of CYP1B1 compared to untreated control samples or LPS-treated control samples for co-treatment are shown as Log2(FC) over time. Macrophages were stimulated with 100 ng/mL LPS or the AhR-ligands BaP (2 µM), I3AA (50 µM), and IAld (50 µM) with or without additional LPS for 2, 4, 16 and 24 h. (**C**) Relative protein abundances of AhR compared to untreated control samples or LPS-treated control samples for co-treatment are shown as Log2(FC) over time. Macrophages were stimulated the AhR-ligands BaP (2 µM), I3AA (50 µM), and IAld (50 µM) with or without additional 10 ng/mL LPS for 2, 4, 16 and 24 h. The significances were determined by student’s *t*-test (*n* = 6 for 2 h and 4 h, *n* = 5 for 16 h and 24 h). (* *p* ≤ 0.05, ** *p* ≤ 0.01, *** *p* ≤ 0.001, # below limit of detection for this time point).

The activation of AhR by the different ligands was investigated based on the expression and the protein abundance of the AhR target protein CYP1B1. Notably, the CYP1B1 mRNA expression was 6-fold higher in the presence of the AhR-ligands in combination with LPS than without LPS (Figure 3A). Only BaP-treatment led to a distinct significant increase of CYP1B1 mRNA with and without LPS co-stimulation. In contrast, the indoles only slightly increased the CYP1B1 expression after 6 h. Significant differences were noticed for I3AA without additional LPS but not for IAld.

Based on the proteome data, the protein abundance of CYP1B1 was monitored over 24 h of exposure and changes were calculated as Log2(FC) relative to the corresponding control with or without LPS, respectively (Figure 3B). All three AhR-ligands significantly increased CYP1B1 levels after 2 h and or 4 h of exposure. Thereby, BaP stimulation with and without LPS led to the highest increase of CYP1B1 after 16 h and 24 h. In contrast, the indoles showed higher CYP1B1 protein levels at the early time points. However, under the additional treatment with LPS, the differences in protein abundances were lower and less significant for all investigated AhR-ligands, especially after 2 h and 4 h of treatment (Figure 3B). The rapid degradation of AhR is a hallmark of its activation. Thereby, nuclear translocation and export precede proteasomal degradation [37,38]. Therefore, the protein levels of AhR were time-dependently analyzed to verify the activation of AhR by the used ligands (Figure 3C). All three ligands led to a rapid decrease of AhR levels, resulting in protein amounts below the detection limit after 2 h. However, for IAld and I3AA, the AhR-levels recovered to the levels before stimulation after 4 h and 16 h, respectively, and BaP-treatment led to a significant decrease lasting at least 24 h. These data indicate that all three used chemicals activate AhR, but with different kinetics and especially signal duration.

Next, the same conditions were used to analyze the effects of AhR-activation during LPS stimulation in macrophages on the proteome level. For this purpose, protein extracts were labeled with tandem mass tags and analyzed with LC-MS/MS. Thus, 4096 proteins were identified in total, of which 3504 were quantified in at least three biological replicates.

**Figure 4:**
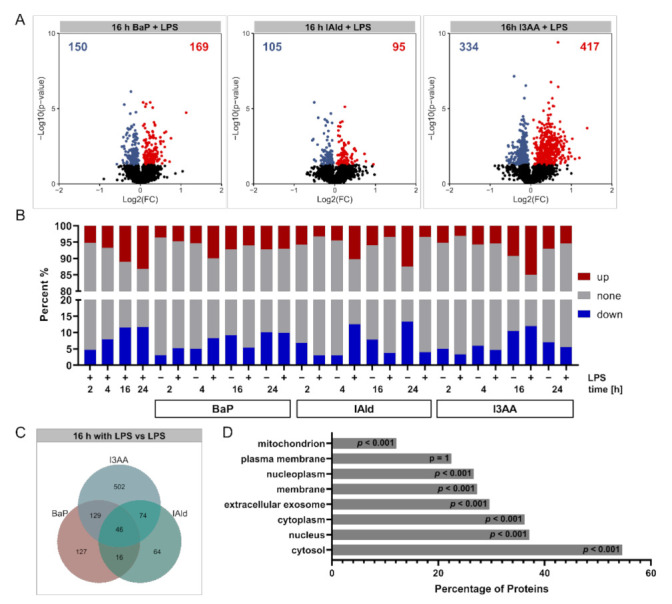
Overview of quantified and regulated proteins. (**A**) Summary of obtained Log2(FCs) and –Log10 (*p*-values) after 16 h of BaP, IAld, or I3AA co-stimulated with LPS compared with the LPS control. Significantly altered proteins were highlighted either blue (*p* ≤ 0.05, Log2 FC < 0) or red (*p* ≤ 0.05, Log2 FC > 0) (*n* = 5). (**B**) Percentages of regulated proteins for all investigated comparisons. Down-regulated fractions (*p* ≤ 0.05, Log2 FC < 0) are shown in blue and up-regulated fractions (*p* ≤ 0.05, Log2 FC > 0) in red for each treatment. (**C**) Overlap of regulated proteins upon co-stimulation with BaP, IAld, or I3AA and LPS after 16 h compared with the LPS control is depicted. (**D**) Mapping of cellular localizations to quantified proteins. Shown are fractions of identified proteins annotated for specific cellular localizations. Significance was calculated by hypergeometric testing with subsequent adjustment for multiple testing, according to Benjamini and Hochberg. Significance of enrichment is provided.

Compared with the respective control samples, significant changes (*p*-value ≤ 0.05, subsequently referred to as regulation) in protein abundances were determined (Supplementary Figure S1, Supplementary Table S1). After 16 h of co-stimulation with BaP, IAld, or I3AA and LPS, high amounts of regulated proteins were detected compared with the LPS control (Figure 4A). While the Log2(FC) ranges for AhR-ligand-treated samples were relatively small, the exposure to LPS with or without AhR led to higher Log2(FC) ranges, indicating more substantial changes (Supplementary Figure S1). Furthermore, the number of regulated proteins increased over time for all conditions. Notably, the fewest changes were observed for the co-treatment with IAld and LPS for all time points (Supplementary Figure S1). 

Furthermore, the fractions of regulated proteins were determined for all treatments (Figure 4B). Depending on stimulation and incubation time, between 6% and 40% regulated proteins were identified. Only a few protein regulations occurred after 2 h and 4 h, whereas the number of altered proteins increased after 16 and 24 h in most conditions. An exception was IAld with LPS co-stimulation that resulted in most regulated proteins after 4 h (about 25%) and considerably less regulated proteins (about 7%) after 16 h and 24 h of co-stimulation. The overlap between regulated proteins obtained for the three AhR-ligands with and without LPS over all time points was relatively low, and most of the regulated proteins were unique for the distinct treatments (Figure 4C, Supplementary Figure S2). The cellular distribution of the identified proteins was examined to confirm that a global proteomic analysis was conducted. This analysis revealed a wide range of cellular localizations. Besides cytosolic, nucleic and membrane proteins were quantified to a comprehensive degree, while plasma membrane-localized proteins were slightly underrepresented (Figure 4D).

**Figure 5:**
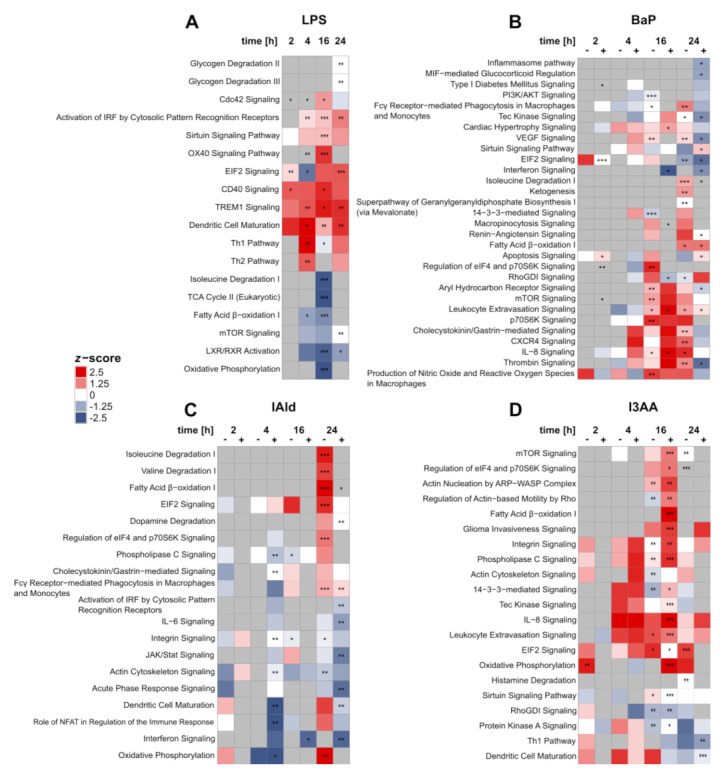
Changes in IPA core pathways over time after treatment with LPS (**A**), BaP (**B**), I3AA (**C**), and IAld (**D**), each with (+) or without (–) LPS co-stimulation and compared with the corresponding control samples (either with or without LPS). Regulation of pathways was identified by *z*-scores. Regulated pathways were marked either red (up, *z*-score > 0) or blue (down, *z*-score < 0). The significance of enrichment was determined by Fisher’s Exact *t*-test, Benjamini-Hochberg adjusted, and indicated with asterisks: adjusted *p*-value ≤ 0.05 (*); adjusted *p*-value ≤ 0.01 (**); adjusted *p*-value ≤ 0.001 (***) (*n* = 6 for 2 h and 4 h, *n* = 5 for 16 h and 24 h).

Next, pathway analysis was conducted utilizing Ingenuity Pathway Analysis (IPA) to determine the cellular effects of AhR activation by BaP, IAld, and I3AA in LPS-stimulated macrophages (Supplementary Table S1). Pathways were considered significantly enriched with an adjusted *p*-value ≤ 0.05 (Supplementary Table S2). As expected, LPS treatment led to a significant increase in proteins associated with immunoregulatory pathways at all time points (Figure 5A). For example, the proinflammatory TREM1 Signaling and the Activation of IRF by Cytosolic Pattern Recognition Receptors were significantly increased after 4, 16, and 24 h. After 2 and 16 h, a significant up-regulation of CD40 Signaling was observed. In contrast, TCA Cycle II was significantly decreased after 16 h of stimulation.

Stimulation with BaP with or without LPS led to the regulation of various cellular processes (Figure 5B). Proteins assigned to AhR signaling, e.g., CYP1B1 or AhR itself, were significantly regulated after BaP treatment (Supplementary Figure S3), indicating this pathway to be affected as well. However, significant enrichment of AhR Signaling was only identified after 16 h of co-stimulation and 24 h without LPS (Figure 5B). While after 16 h BaP treatment AhR Signaling was found up-regulated, it was inhibited after 24 h of BaP and LPS co-treatment compared with LPS alone. Furthermore, after 24 h of co-stimulation with BaP and LPS, a significant down-regulation of the Inflammasome pathway was detected. The Production of Nitric Oxide and Reactive Oxygen Species in Macrophages was significantly up-regulated after 16 h of BaP treatment without endotoxin. The same was noticed as a trend at all other time points without additional LPS and after 16 h of co-stimulation.

The treatment with IAld generally led to the fewest changes on pathway level (Figure 5C). As a significantly decreased process, Acute Phase Response Signaling was identified after 24 h of co-stimulation. Additionally, significantly up-regulated pathways like Fatty Acid β-oxidation I or Regulation of elF4 and p70S6K Signaling were found after 24 h of IAld-stimulation, only without LPS. One exception is Fcγ Receptor-mediated Phagocytosis in Macrophages and Monocytes, which was found up-regulated for IAld and LPS co-stimulated cells likewise.

Up-regulated processes were prevalent for the stimulation with I3AA (Figure 5D), which were mainly significant after 16 h of incubation, independent of LPS stimulation. One down-regulated process is RhoGDI Signaling, which was significantly downregulated after 16 h, both after stimulation with I3AA alone and co-stimulation with LPS.

Especially metabolic pathways were regulated frequently. For instance, Fatty Acid β-oxidation I was significantly decreased after 4 and 16 h of LPS stimulation. In contrast, this process was significantly increased after incubation with the AhR-ligands (Figure 5A–D). After 24 h of BaP treatment, this metabolic process was significantly enriched, independent of LPS co-stimulation. Furthermore, Fatty Acid β-oxidation I was significantly increased after 24 h of IAld stimulation without LPS and likewise after 16 h of co-stimulation with I3AA and LPS. In LPS-treated macrophages, a significant reduction of Oxidative Phosphorylation was noticed after 16 h and after 4 h of co-stimulation with IAld and LPS (Figure 5A). The same trend was found without LPS co-stimulation. In contrast, 24 h of IAld treatment led to an up-regulation of Oxidative Phosphorylation (Figure 5C). Due to stimulation with I3AA, this process was significantly up-regulated after 2 h and after 16 h of co-stimulation with LPS (Figure 5D). The same trend can be seen after 24 h of incubation with the ligands alone. Furthermore, amino acid metabolism was regulated by BaP, IAld, and LPS, but not I3AA (Figure 5A–D). 

In general, similar metabolic pathways were regulated by most of the used chemicals. Whereas under LPS stimulation, a down-regulation after 16 h was observed, and the AhR-ligands led to an up-regulation. Nevertheless, also time- and ligand-dependent effects were detected. IAld up-regulated metabolic processes after 24 h, but I3AA showed significant increases after 16 h of co-stimulation with LPS compared with LPS only. The metabolic impact of BaP was more time-dependent than for the indoles. In the range from 16 to 24 h, an up-regulation with and without additional LPS stimulation was observed.

Additionally, the regulation of mTOR Signaling and its downstream process Regulation of elF4 and p70S6K Signaling should be noticed, since they are linked with key pathways in metabolism and the immune response in macrophages [39,40]. In LPS-treated cells, a slight but not significant down-regulation of mTOR Signaling was observed after 4 h and 16 h. In contrast, BaP treatment for 16 h led to a significant increase of mTOR Signaling, p70S6K Signaling, and Regulation of eIF4 and p70S6K Signaling. The same trend was observed after 16 h of co-stimulation and 24 h of BaP treatment. At the last time point of IAld stimulation without LPS, the Regulation of eIF4 and p70S6K Signaling was also up-regulated. After 16 h of co-stimulation with I3AA and LPS, mTOR Signaling and the Regulation of eIF4 and p70S6K signaling were significantly up-regulated.

Besides metabolic, also immunoregulatory processes were affected by the three AhR-ligands (Figure 5B–D). The stimulation with BaP only led to a significant up-regulation of Fcγ Receptor−mediated Phagocytosis in Macrophages and Monocytes after 24 h. This process was affected likewise after 24 h of treatment with IAld, independent of the presence of LPS. Interferon Signaling was significantly down-regulated after 16 h and 24 h of co-stimulation with BaP or IAld and LPS. In contrast, this process was not affected by I3AA. Furthermore, changes in interleukin signaling were observed for BaP, IAld, and I3AA (Figure 5B–D). IL-6 signaling was significantly decreased after 24 h of co-stimulation with IAld and LPS, whereas IL-8 signaling was significantly up-regulated after BaP and I3AA treatment. The treatment with BaP without LPS for 16 and 24 h and the co-stimulations with BaP or I3AA and LPS for 16 h significantly up-regulated IL-8 Signaling. Additionally, the same increasing trend was detected for I3AA treatment over all time points except 2 h, independent of LPS co-stimulation. After 4 h of incubation with BaP, this upregulation occurred only in combination with LPS. In summary, various effects on immunoregulatory processes were observed after treatment with the AhR-ligands, depending on LPS co-stimulation. While some processes were decreased, as the Inflammasome pathway, Interferon Signaling or IL-6 signaling, and others, like Fcγ receptor-mediated phagocytosis in macrophages and monocytes or IL-8 signaling, were up-regulated.

Taken together, time- and LPS-dependent effects of the AhR agonists BaP, IAld, and I3AA on the proteome of activated macrophages were observed. Additionally, stimulation of endotoxin-activated macrophages with the AhR-ligands led to ligand-specific regulation of metabolic and immunoregulatory processes. The stimulation with all three ligands resulted in an up-regulation of metabolic processes as Oxidative Phosphorylation, Fatty Acid β-oxidation I, or amino acid degradation. Furthermore, different immunoregulatory processes were affected by the chemicals. Thereby, BaP-stimulation led to most changes in these pathways, including the decrease of proteins associated with the Inflammasome pathway.

## 4. Discussion

The AhR has important immunomodulatory properties, such as limiting the release of proinflammatory cytokines and protection from septic shocks [7,24,25]. Thereby, different tryptophan-derived AhR-ligands like I3AA and indole-3-lactic acid attenuate the immune response in macrophages after stimulation with LPS [41,42]. In this study, the down-regulation of TNF expression in primary human macrophages due to co-stimulation with LPS and the AhR-ligands IAld and I3AA was shown, but no regulatory effects were found on TNF secretion (Figure 2). These results are similar to recent findings, where the AhR-ligands BaP and FICZ had no significant effect on TNF secretion in co-stimulated human macrophages over time, except for the earliest time point. However, they also found the mRNA levels of TNF significantly decreased due to co-stimulation [27]. For primary human macrophages, this suggests the AhR-dependent regulation of TNF expression in the presence of LPS, at least after 6 h of incubation, with no effect on TNF secretion at later time points (16–24 h). Thus, besides a temporal dependency, specific regulation of the TNF release may depend on the macrophage subtype and the AhR-ligand. Notably, especially time-dependent data on the effect of AhR and TLR4 co-stimulation of primary human macrophages have been scarce so far. Therefore, this study aimed to time-dependently characterize AhR-dependent cellular processes in human primary macrophages through quantitative proteomics. For this purpose, the ligands BaP, IAld, and I3AA were investigated with and without LPS co-stimulation for up to 24 h. 

The activation of AhR was verified by the protein abundance and the expression level of its target protein CYP1B1, which is the predominant form of P450 in blood-derived monocytes and macrophages [43]. Thus, we found increases in CYP1B1 levels already after 2 h, which further increased at the later time points for BaP but not in the presence of the indoles (Figure 3). In addition, AhR activation was confirmed by monitoring the degradation of AhR after stimulation, which is a hallmark for completed AhR nuclear import and export [37,38].

The degradation of BaP by CYP1B1 is up to three times slower than the metabolization by CYP1A1 [44]. This enzyme-dependency might lead to a prolonged AhR activation by BaP compared with the indoles [45], which is in line with the results observed herein. In contrast, IAld is rapidly degraded by aldehyde dehydrogenase [46,47]. In addition, the indoles might not be as potent AhR agonists as BaP, but they are relevant molecules which can affect microbiome homeostasis and host immune response [48].

Based on the proteome, affected pathways and cellular processes were identified. As suspected, proinflammatory pathways like TREM1 Signaling and the Activation of IRF by Cytosolic Pattern Recognition Receptors [49,50,51] were up-regulated by treatment with the endotoxin LPS (Figure 5A). A drastic switch of the metabolism usually follows the activation of macrophages. In proinflammatory macrophages, the up-regulation of glycolysis and the pentose phosphate pathway, as well as the down-regulation of the TCA cycle, oxidative phosphorylation, and fatty acid β-oxidation, were described [52,53]. These decreases in metabolic processes were found in our results upon LPS treatment as well, confirming the appearance of proinflammatory processes due to LPS.

### 4.1. Anti-Inflammatory Modulation of Cell Metabolism in LPS-Activated Macrophages through AhR-Ligands

The AhR-ligands BaP, IAld, and I3AA affected metabolic as well as immunoregulatory processes in the investigated human macrophages with or without additional LPS stimulation. In most cases, pathways like oxidative phosphorylation, fatty acid β-oxidation, or amino acid degradation were up-regulated, opposite to the LPS-induced effects. Furthermore, immunological processes were regulated in two different ways. Phagocytic processes were increased by BaP and IAld, and BaP as well as I3AA increased IL-8 signaling. A reduction of the Inflammasome pathway by BaP and Interferon Signaling by BaP and IAld was observed (Figure 5B–D). Previous studies have shown that metabolic processes and macrophage functions are closely linked [52]. In proinflammatory macrophages, suppression of oxidative phosphorylation was observed, leading to ROS production in the lysosome. On the other hand, anti-inflammatory macrophages show enhanced oxidative phosphorylation, and the M2 polarization is dependent on oxidative phosphorylation but not on glycolysis [54,55]. These studies suggest that anti-inflammatory processes in macrophages are linked to enhanced oxidative phosphorylation compared with proinflammatory immune responses. This tendency can be seen in our results as well, especially after 16 h of co-stimulation with I3AA and LPS compared with the activation with LPS, where this pathway was significantly increased (Figure 5D).

Additionally, fatty acid oxidation affects immune processes and has been described to be decreased in proinflammatory macrophages [56,57]. Enhancement of this reaction in palmitate-treated RAW 264.7 macrophages reduced the inflammatory reaction and cytokine production [58]. Here, we found that fatty acid β-oxidation I was significantly increased by all three AhR-ligands after 16 or 24 h of treatment, dependent on LPS co-stimulation, suggesting the anti-inflammatory potential of the here tested AhR-ligands in human macrophages.

### 4.2. Potential Role of mTOR Signaling in AhR-Dependent Immunomodulation

Further, the mTOR Signaling pathway and the downstream processes p70S6K and eIF4 Signaling were up-regulated by the three AhR-ligands (Figure 5B–D). On the one hand, mTOR Signaling depends on high cellular ATP levels [59,60], which may be linked to the AhR-dependent up-regulation of oxidative phosphorylation and metabolism in general. On the other hand, mTOR acts as an anti-inflammatory signaling pathway necessary for the IL-4-dependent polarization of anti-inflammatory M2 macrophages [61,62]. Furthermore, mTOR signaling is activated in macrophages by LPS via the PI3K/AKT-pathway, thus regulating the activity of NF-κB and the production of immunoregulatory cytokines such as IL-10, TNF, and IL-12 [40]. Inhibition of mTOR complex 1 (mTORC1) in endotoxin-activated macrophages is followed by an enhanced NF-κB activity as well as IL-12 and TNF production, but a decreased IL-10 production [63]. Consequently, the here-observed up-regulation of the mTOR pathway as well as p70S6K and eIF4 signaling in co-stimulated cells may lead to a suppression of the LPS-dependent immune-response, as indicated by the increased fatty acid β-oxidation I.

Additionally, Saric et al. showed that mTOR controls the reformation of mature lysosomes (tubulation) in RAW 264.7 macrophages via mTORC1 and the PI3K/AKT-Pathway, which is necessary to activate phagocytosis [64]. An enhanced proteomic ability for phagocytosis was also detected in our data (Figure 5B,C).

As recently shown, the activation of AhR by BaP in LPS-treated blood-derived human macrophages is followed by a decreased activity of AKT and a reduced TNF secretion compared with LPS after 2 h, which shows the complex impact of AKT on the regulation of the immune response. In line with this, AKT interacts with mTROC1 and mTORC2 and is activated by various pathways [65]. We observed an up-regulation of mTOR Signaling-associated proteins due to AhR activation. Thus, our proteome data is in accordance to the before-described immunoregulatory effects of these processes. This suggests an AhR-dependent enhanced impact of mTOR signaling and associated pathways in feedback regulation of the immune response in endotoxin-activated macrophages.

### 4.3. Ligand-Specific Effects on Pathways of the Immune Response

In this study, immune-regulatory pathways like Fcγ receptor-mediated phagocytosis and Nitric Oxide and ROS production were found up-regulated at the protein level upon stimulation with the AhR-ligands, especially BaP and IAld (Figure 5B,C). Fueldner et al. [26] showed that pre-treatment of murine bone marrow-derived macrophages (BMM) with BaP and subsequent stimulation with a broad spectrum of pathogen-associated molecular patterns led to an increase of phagocytotic processes in an AhR-dependent manner. Besides a higher uptake rate per cell, the synthesis of NO was enhanced [26], which is in line with our results. These results suggest an increase of phagocytosis by AhR-activation in co-stimulated macrophages, as indicated by the increased mTOR processes.

**Figure 6:**
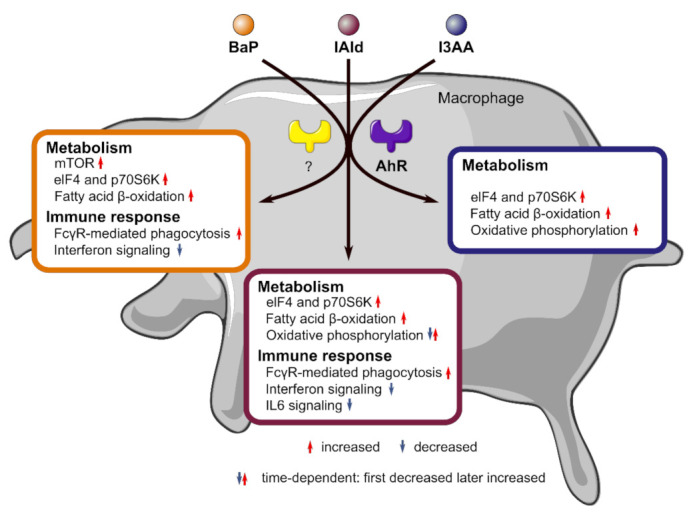
Overview on affected metabolic and immunological key pathways in macrophages due to stimulation with the AhR ligands BaP, IAld, and I3AA with or without LPS co-stimulation. Metabolic pathways like mTOR, fatty acid β-oxidation, or oxidative phosphorylation as well as immune regulatory pathways like Fcγ receptor (FcγR)-mediated phagocytosis or interferon signaling were regulated in a ligand-dependent manner.

Here, the Inflammasome pathway and IL-6 signaling were down-regulated by BaP or IAld co-stimulated with LPS. In contrast, IL-8 Signaling was up-regulated by BaP and I3AA in combination with LPS. Analogous to these results, Ishihara et al. found a varying effect of the AhR-ligands TCDD, FICZ, and indole-3-carbinol (I3C) in LPS-treated murine BMM. The expression of C-C motif chemokine 2 (CCL2), IL-6, and IL-10 in LPS-treated BMMs was induced by TCDD and FICZ but suppressed by I3C [66]. These results suggest a ligand-dependent regulation of the immune response in LPS-stimulated murine macrophages due to AhR activation. The duration of AhR activation and thus the stability of AhR-ligands is suggested to be a primary factor for AhR ligand-specific effects [67]. This might contribute to the observed differences, especially between BaP and the indoles analyzed, and needs further investigation considering the constant replenishment of microbial tryptophan metabolites in the intestine. Distinct effects of different AhR-ligands were also shown for the non-genomic immunoregulatory activity of the receptor in human macrophages. While BaP exposure of endotoxin-activated macrophages led to an inhibition of Rac1-signaling and AKT-activity, FICZ seemed not to suppress these pathways. However, stimulation with both molecules was followed by the same ratio of decreased AKT phosphorylation [27]. Even the structurally similar tryptophan metabolites IAld and I3AA affected the immune response in RAW 264.7 macrophages activated with LPS in different ways [48]. Whereas treatment with 500 µM IAld for 2 h was followed by a distinct reduction of IL-1β, IL-6, and TNF, only slight changes of IL-1β and TNF expression were observed after stimulation with the same amount of I3AA [48]. In this study, a 10-fold lower concentration (50 µM) of the respective indole metabolites was used and had cellular effects in human macrophages already. Our data showed the same amount of significant mRNA reduction after IAld and I3AA simulation in LPS-treated human macrophages after 6 h (Figure 2). These varying results may either refer to different processes in murine and human macrophages or to dose and time-dependent effects. An overview on affected metabolic and immunological key pathways is provided in Figure 6.

Furthermore, we noticed only a small overlap between regulated proteins due to the three used AhR-ligands (Figure 4C, Supplementary Figure S2). This holds for the time-dependent protein regulation by each ligand, independent of LPS co-stimulation. It is known that different AhR-ligands may have cell-specific, tissue-specific, and ligand-specific effects when they act as selective AhR modulators (SAhRMs) [68]. Kopec et al. tested the dioxin-like AhR agonists TCDD, 3,3′,4,4′,5-pentachlorobiphenyl (PCB126) and 2,3,7,8-tetrachlorodibenzofuran (TCDF) in mouse liver and found between 1411 and 3280 regulated proteins with an overlap of 202 proteins only. Hence, this study suggested that even AhR-binding of structurally similar ligands may lead to different effects on the proteome [69]. Our results support this, because for BaP and especially the used, structurally similar indoles IAld and I3AA, a ligand- and time-dependent protein regulation due to AhR activity in primary human macrophages was observed (Figure 5B,C; Supplementary Figure S2).

Observing ligand-specific effects, potential AhR independent mechanisms should be considered, likewise. In a recent study, Langan et al. demonstrated differing effects for IAld and I3AA [48]. IAld I3AA inhibited the expression of IL1b and IL6 in an AhR dependent manner, whereas I3AA showed AhR-independent anti-angiogenic activity in cell-based models of rheumatoid arthritis [48]. Further, I3AA was shown to neutralize free radicals and thereby mitigate inflammatory responses of RAW264.7 macrophages in response to LPS [70]. In addition, non-classical genomic AhR-signaling differing ligand structure dependently and non-genomic AhR-signaling should be considered [71,72].

In summary, our data suggested ligand-dependent, time-dependent, and LPS-dependent effects of AhR-activation on human macrophages. Further studies have to show how the co-stimulation of endotoxin with the AhR-ligands BaP, IAld, and I3AA regulates the cellular activity of macrophages and how this may be used as a potential therapy for inflammatory diseases.

### 4.4. Potential Role of AhR-Ligands in Gut Microbial Homeostasis

Homeostasis of a healthy gut microbiome requires a balance between proinflammatory processes to fight pathogens and anti-inflammatory processes to protect commensals [73,74]. For this balance, the activation tuning of AhR by microbial tryptophan metabolites is essential [15,74]. In turn, a restricted capability to produce indoles from tryptophan is associated with metabolic syndrome [14], and a complete lack is linked to inflammatory bowels disease [19]. IAld, one of the tryptophan metabolites investigated in this study, has recently been used in the form of encapsulated microparticles to protect mice in a murine metabolic syndrome model [75]. In our study, IAld led to a decreased capability for IL6 signaling and, like BaP and I3AA, reduced the TNF transcription. In contrast, both were enhanced after LPS-treatment of macrophage-specific AhR-KO mice, ultimately leading to septic shock [24,25]. Additionally, we could show that all tested ligands impact fatty acid metabolism, which is in the context of the importance of short-chain fatty acids in maintaining the gut barrier function [73,76]. Thus, the here-shown proteomic changes in response to AhR-activation potentially foster the understanding of ligand-specific AhR-dependent processes in maintaining homeostasis.

## 5. Conclusions

In this study, LPS-activated primary human macrophages were stimulated with the AhR-ligands BaP, IAld, and I3AA for 2, 4, 16, and 24 h. Ligand-dependent effects on regulated proteins were found for all time points. Additionally, canonical pathways were affected in a ligand- and time-dependent manner. An enhancement of metabolic processes in dependency of additional LPS stimulation was observed for all investigated conditions. Furthermore, immunoregulatory pathways were affected by all three AhR-ligands, even though the different ligands led to distinct regulations. Nevertheless, the consistent up-regulation of metabolic processes due to AhR-activation as well as the suppression of several immunological pathways on the proteomic level indicated an AhR-dependent inhibition of proinflammatory signals in human macrophages, which may be involved in maintaining microbial homeostasis.

## Data Availability

The proteomics data have been deposited to the ProteomeXchange Consortium via the PRIDE partner repository with the dataset identifier PXD025022.

## Supplementary Materials

The following are available online at https://www.mdpi.com/article/10.3390/ijerph181910336/s1, Table S1: Fold Changes for all replicates and stimulation time points with results of statistical analyses, Table S2: IPA results for core pathways, shown as zscores and log10 (*p*-values), Figure S1: Changes in protein abundances after 2, 4, 16, and 24 h of BaP, IAld, or I3AA treatment with or without LPS stimulation compared to the respective control, Figure S2: Overlap of regulated proteins, determined in comparison to the respective controls, after 2, 4, 16, and 24 h of stimulation with BaP, IAld, or I3AA with or without LPS, Figure S3: Simplified genomic AhR-Pathway based on the IPA database.
