# Supplementary material for: Proteomic Characterization of the Cellular Effects of AhR Activation by Microbial Tryptophan Catabolites in Endotoxin-Activated Human Macrophages
Source: Int J Environ Res Public Health. 2021 Sep 30;18(19):10336. doi: 10.3390/ijerph181910336 (PMC8507890; doi:10.3390/ijerph181910336)
Supplement: Supplementary file 1 [file ijerph-18-10336-s001.zip › ijerph-1374965-supplementary/ijerph-1374965-supplementary-xml conversion/Supplementary Figures S1-S3.pdf]

International Journal of
Environmental Research and Public Health

MDPI

Supplementary Figures

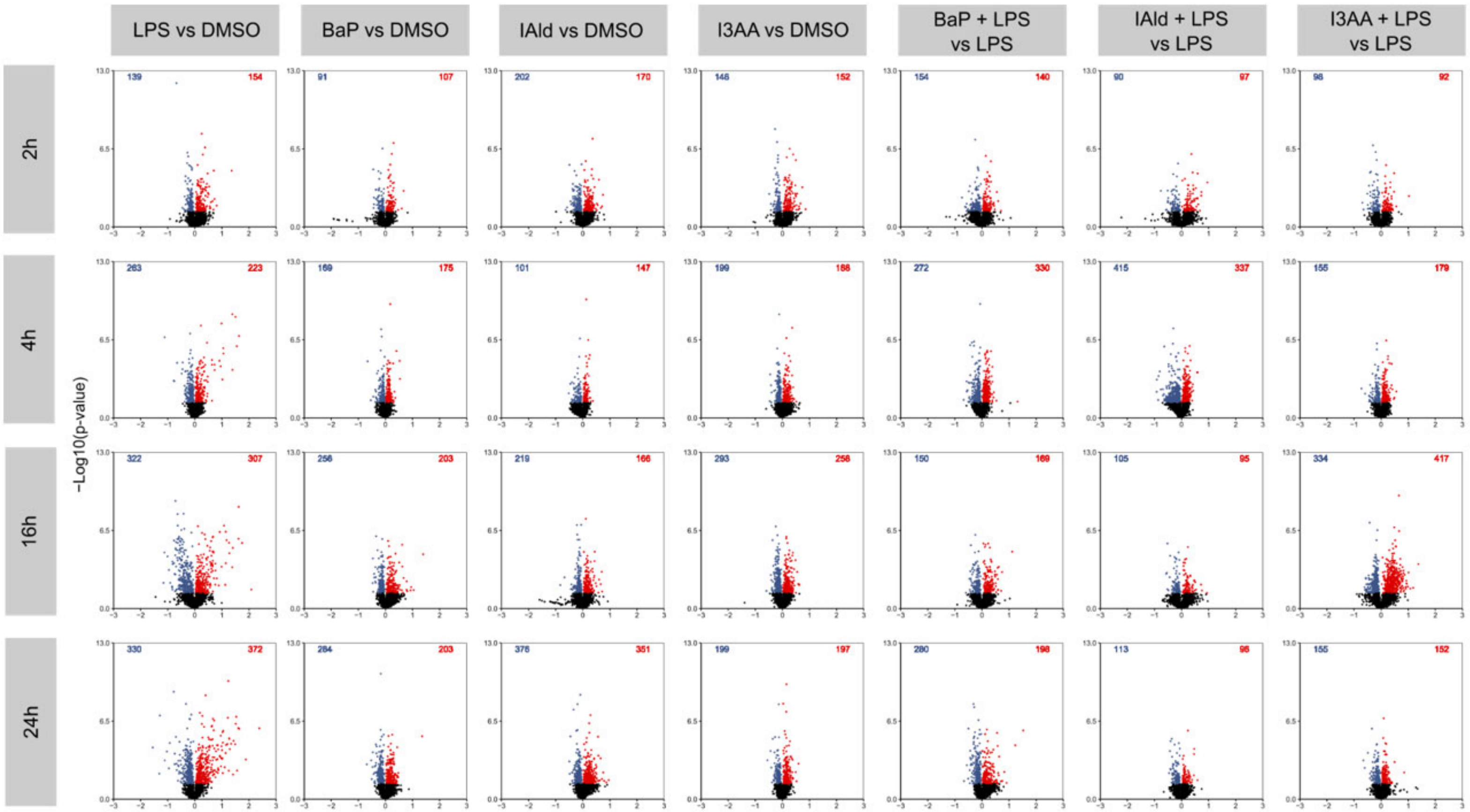

Figure S1. Changes in protein abundances after 2, 4, 16, and 24 h of BaP, IAld, or I3AA treatment with or without LPS stimulation compared to the respective control. Significantly affected proteins (regulated proteins) were highlighted in blue ($p \leq 0.05$, Log2 FC < 0) or red ($p \leq 0.05$, Log2 FC > 0) ($n = 6$ for 2 h and 4 h, $n = 5$ for 16 h and 24 h).

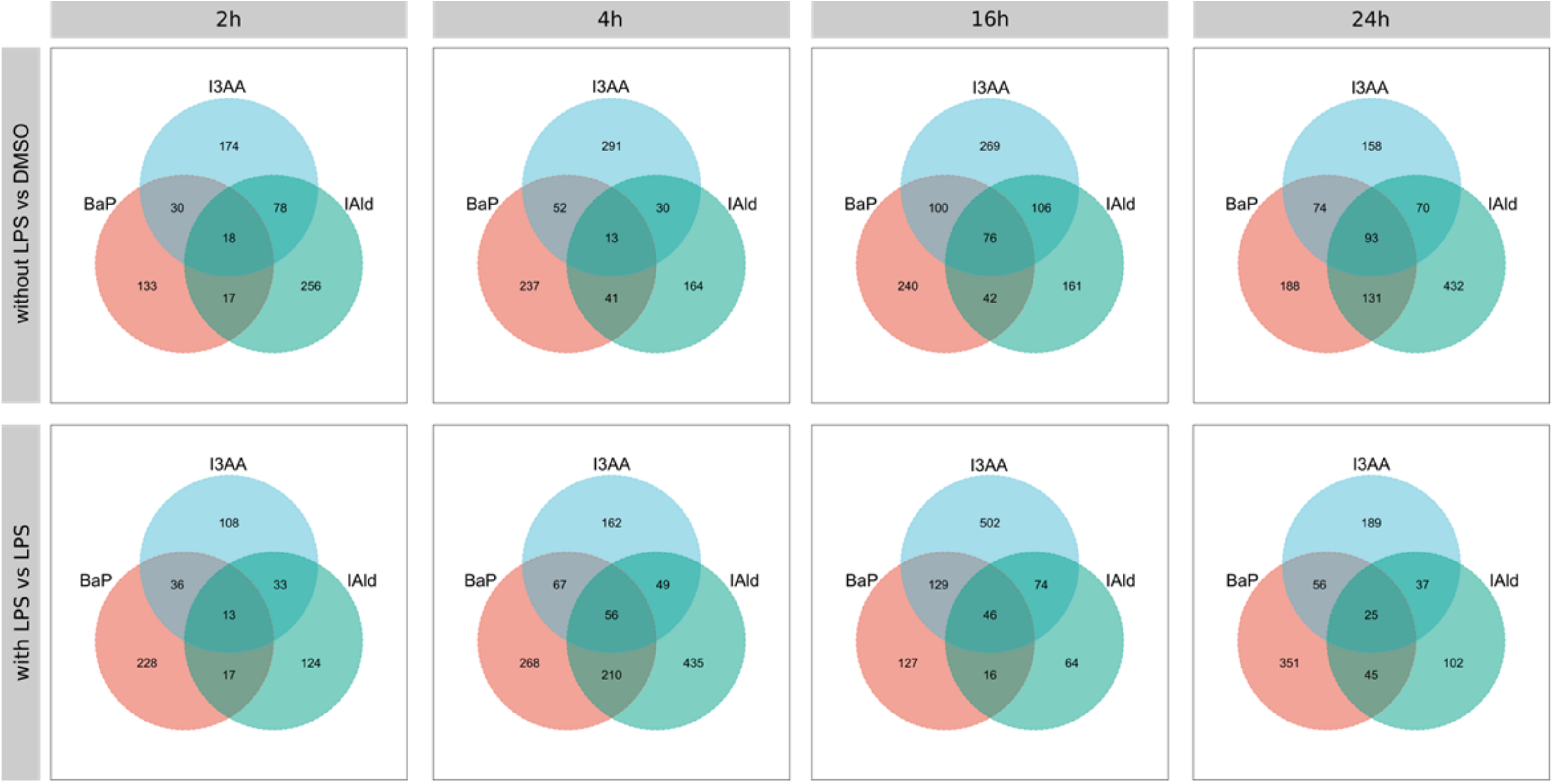

Figure S2. Overlap of regulated proteins, determined in comparison to the respective controls, after 2, 4, 16, and 24 h of stimulation with BaP, IAld, or I3AA with or without LPS (n = 6 for 2 h and 4 h, n = 5 for 16 h and 24 h).

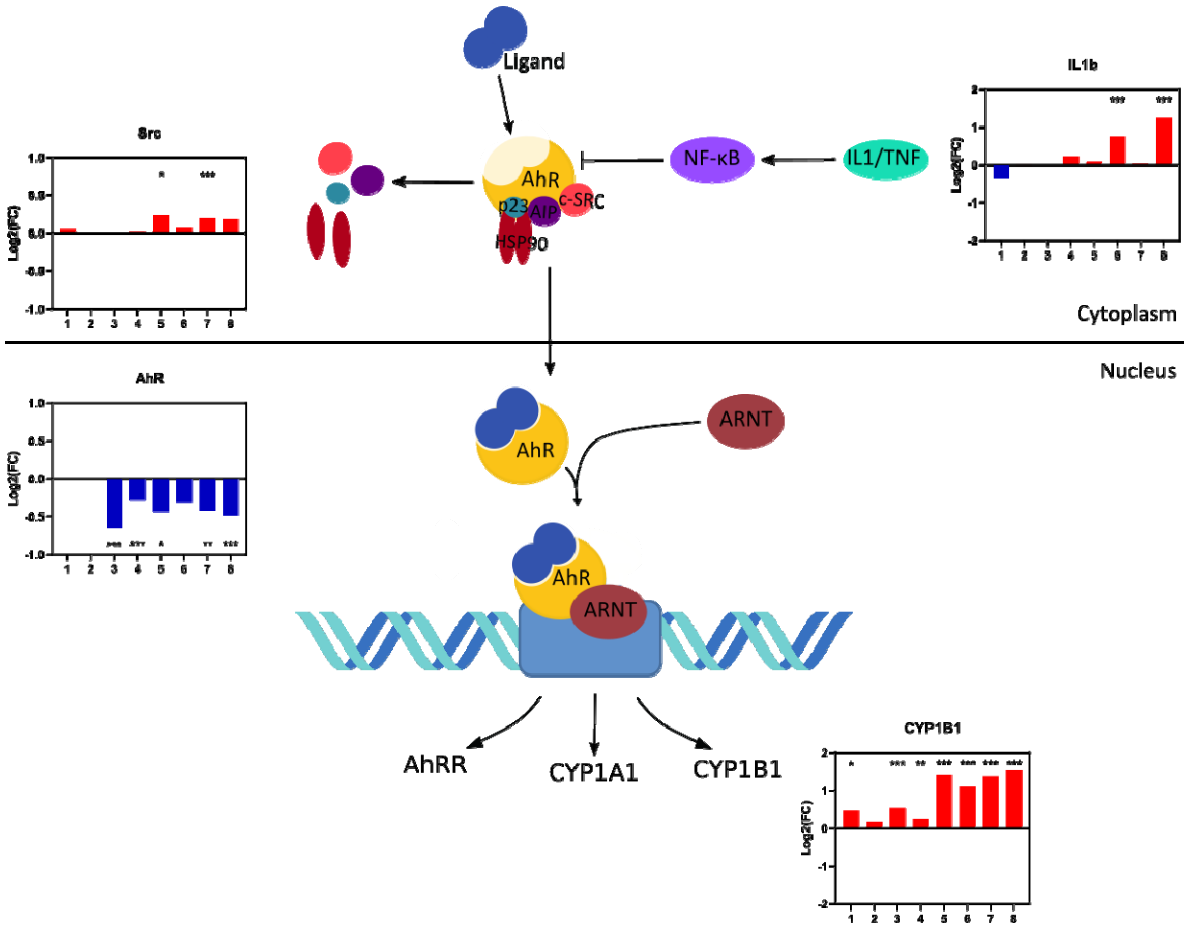

Figure S3. Simplified genomic AhR-Pathway based on the IPA database. AhR is released from the cytosolic multi-protein complex after ligand binding and moves to the nucleus. There, a complex with the AhR nuclear translocator (ARNT) is formed to enable DNA-binding and transcription of target genes as the AhR repressor (AhRR) or Cytochrome P450 (CYP) 1A1 and 1B1. Activation of the NF-κB pathway, e.g. by IL-1β or TNF, can inhibit AhR-activity. The detected abundances of regulated proteins related to this pathway are shown for stimulation with BaP as blue (Log2 FC < 0) or red (Log2 FC > 0). The significances are indicated with asterisks: $p \leq 0.05$ (*), $p \leq 0.01$ (**), $p \leq 0.001$ (***). The different conditions are numbered as follows: 1 = 2 h BaP vs DMSO; 2 = 2 h BaP + LPS vs LPS; 3 = 4 h BaP vs DMSO; 4 = 4 h BaP + LPS vs LPS; 5 = 16 h BaP vs DMSO; 6 = 16 h BaP + LPS vs LPS; 7 = 24 h BaP vs DMSO; 8 = 24 h BaP + LPS vs LPS (n = 6 for 2 h and 4 h, n = 5 for 16 h and 24 h).
